# A Sustainable Approach to Phosphorus Nutrition in Banana Plantations

**DOI:** 10.3390/plants14131923

**Published:** 2025-06-23

**Authors:** Hebert Teixeira Cândido, Magali Leonel, Sarita Leonel, Adalton Mazetti Fernandes, Jackson Myrellis Azevêdo Souza, Lucas Felipe dos Ouros, Paulo Ricardo Rodrigues de Jesus

**Affiliations:** 1 Capixaba Institute for Research, Technical Assistance and Rural Extension (INCAPER), Atílio Vivácqua 29.490-000, ES, Brazil; hebert.candido@incaper.es.gov.br; 2 São Paulo State University (UNESP), Center for Tropical Roots and Starches (CERAT), Botucatu 18.610-034, SP, Brazil; lucas.ouros@unesp.br; 3 São Paulo State University (UNESP), School of Agriculture (FCA), Botucatu 18.610-034, SP, Brazil; sarita.leonel@unesp.br (S.L.); pr.jesus@unesp.br (P.R.R.d.J.); 4 Federal University of Viçosa (UFV), Department of Agronomy, Viçosa 36.570-900, MG, Brazil; jackson.m.souza@ufv.br

**Keywords:** *Musa* spp., thermophosphate, mineral nutrition, bunch mass, soil, leaf

## Abstract

The genetic diversity of banana plants (*Musa* spp.) can result in different phosphorus requirements, highlighting the importance of studies performed to optimize phosphate fertilization in order to improve the productivity and sustainability of banana plantations. This study assessed the effects of phosphate fertilization on the duration of the harvest season, bunch mass, soil fertility and foliar nutrition of BRS SCS Belluna banana plants. A replicated trial was performed in two consecutive harvests, with different phosphorus levels, i.e., 25, 50, 75, 100, 125 and 150% of the recommended level for the crop. Soil analyses included macro- and micronutrients, silicon, acidity, organic matter, cation exchange capacity and base saturation. Leaf tissue was analyzed for mineral content. Thermophosphate had different effects on soil fertility and leaf nutrients. Calcium and phosphorus in the soil increased linearly. In the leaf, a reduction in zinc content was mainly observed. The lower temperatures and accumulated rainfall that occurred during the second harvest season are related to a greater number of days between flowering and harvest and a lower bunch mass. These results could support fertilization programs aimed at ensuring the long-term sustainability of phosphorus nutrition in banana plantations.

## 1. Introduction

Phosphorus (P) is one of the macronutrients necessary for plant development, with crucial roles in plants’ primary metabolism, such as in photosynthesis, respiration and energy transfer, so alongside nitrogen, it is recognized as a major limiter for agricultural crops [1]. Due to its importance to life, there is a global concern about the levels of its reserves, which, although the expectation of exploitation has increased for another 300–400 years, are still finite and are concentrated in a small number of countries [2,3]. This scenario could compromise production in countries where agriculture plays a key role in the gross domestic product and which depend on imports of this mineral to meet their demand, as is the case in Brazil [4,5].

Low P availability is a global problem that limits yields [6]. Phosphate fertilizer faces the challenge of low efficiency in phosphorus absorption by plants [2]. Although total phosphorus levels in the soil vary from 200 to 3000 mg kg^−1^, less than 0.1% of this is available for absorption, falling between 0.002 and 2.0 mg dm^−3^. This availability is influenced by soil pH, with maximum availability occurring at pH 6.0, in the predominant form of H_2_PO_4_^−^. In acidic soils, common in tropical and subtropical regions, phosphorus can be fixed by aluminum or iron oxides, becoming unavailable to plants [3].

The banana agribusiness is an important sector in the world and in Brazilian agriculture, and its cultivation can be improved with the application of phosphorus, which enhances its productive and nutritional properties. The rational use of correctives and fertilizers in banana plantations depends on reliable standards for interpreting soil fertility. A banana grower can suppress doses of P on a one-off and temporary basis in areas of accumulated fertility with proper productivity monitoring and soil and leaf analysis [7,8,9,10].

Thermophosphates are a source of P, and the production process is based on heating the phosphate rock. The gradual release of P from thermophosphates is promoted by the natural acidity of the soil solution or by the influence of roots and can improve the chemical conditions of the soil, such as pH, base saturation, and the concentration of minerals such as phosphorus, silicon, calcium and magnesium. The characteristics of this fertilizer result in a lower P fixation rate than that of other sources, due to its lower P_2_O_5_ solubility, competition with anionic calcium and magnesium silicates, and increased soil pH [4,11,12].

The benefits provided by thermophosphate may be advantageous for banana plants. These plants absorb large amounts of cations, mainly K^+^ and NH4^+^, and due to their high rate of biomass production, they release large amounts of H^+^ into the rhizosphere, lowering the pH of this region. This acidification increases the levels of toxic aluminum (Al^3+^) in the soil solution. In addition to the immobilization of phosphorus, it has already been shown that Al^3+^ impairs the absorption of water and nutrients by banana plants, compromising their growth and chemical composition [13,14].

Furthermore, it is worth mentioning the great genetic diversity of banana plants; each genotype has different nutritional requirements, which should be investigated [15]. The development of banana cultivars with effective mechanisms for Pi uptake and Pi homeostasis under conditions of P limitation is important to reduce the use of P fertilizers and increase agricultural sustainability [6,8].

The cultivar BRS SCS Belluna is a triploid genotype of the species *Musa acuminata* (genomic group AAA) developed by the Genetic Breeding Programs of the Brazilian Agricultural Research Corporation (EMBRAPA) and the Agricultural Research and Rural Extension Corporation of the State of Santa Catarina (EPAGRI) and launched in 2016. The cultivar is resistant to yellow sigatoka (*Mycosphaerella musicola*, Leach) and Fusarium wilt disease (*Fusarium oxysporum* f. sp. cubense) and moderately resistant to black sigatoka (*Mycosphaerella fijiensis*, Morelet), the main diseases causing damage to banana growing in Brazil and worldwide. This cultivar is therefore a promising option for diversifying banana plantations [16,17]. The plant is 1.72 m tall on average, with bunches weighing 8.2 kg, a rachis mass of 626.5 g, a fruit mass of 5.52 kg, a number of fruits per bunch of 81.2 and 7.4 hands per bunch. The second hand of the bunch has an average of 1022.7 g and 14.5 fruits, with a length of 13.2 cm, a diameter of 36.2 mm and an average fruit weight of 78.5 g each. A ripe banana has a soluble solid content of 24.65 °Brix, a titratable acidity of 0.66 g 100g^−1^ of malic acid and a ripeness index or ratio of 36.15. Green bananas have a starch content of 78.5 g/100 g, of which 51.4 g/100 g is resistant starch (73.7% of the total starch content) [17]. They are suitable for fresh and processed consumption, especially as flour and paste (dehydrated bananas) [17,18].

This research is based on the prevalence of phosphorus binding in tropical soils, the characteristics of thermophosphate fertilizers and the importance of adapting fertilization programs according to genotype with the aim of expanding banana cultivation in Brazil.

Considering the hypothesis that the application of thermophosphate in crop cycles can improve soil fertility and the nutritional status of banana plants, this study aimed to assess the effect of applying various amounts of thermophosphate to BRS SCS Belluna banana plants on soil chemistry, foliar nutrition and the relationship between these factors and crop duration and yield.

## 2. Results

### 2.1. Chemical Soil Properties

Fertilization with thermophosphate at different times and growing seasons on BRS SCS Belluna banana plants showed nutrient-dependent effects on soil fertility attributes. Fertilizing with thermophosphate did not change soil fertility attributes (Table 1) after the following fertilization steps: planting, formation, first fertilization (2020/21) and second fertilization (2021/22). Furthermore, the variability of the response was not explained by the regression analysis, as the adjustments to the curves were low and not significant: organic matter (OM) (R^2^ linear = 0.2153; R^2^ quadratic = 0.2281), pH (R^2^ linear = 0.3294; R^2^ quadratic = 0.3606), Al^3+^ (R^2^ linear = 0.0852; R^2^ quadratic = 0.0902), H (R^2^ linear = 0.0878; R^2^ quadratic = 0.5009), cation exchange capacity (CEC) (R^2^ linear = 0.0704; R^2^ quadratic = 0.3972) and base saturation (BS) (%) (R^2^ linear = 0.4341; R^2^ quadratic = 0.4341).

No differences were observed between the means of macronutrients in the soil depending on the application of thermophosphate (Table 2 and Table 3). Additionally, the regressions were not significant for K (R^2^ linear = 0.0204; R^2^ quadratic = 0.2429) and S (R^2^ linear = 0.0451; R^2^ quadratic = 0.0524) and did not suggest a decrease in the concentration of these minerals in the soil solution.

There is no evidence that fertilization contributed to the immobilization of P released by thermophosphate, as indicated by the lack of influence on P_total_ and the estimate of unavailable phosphorus (P_total_ − P_resin_) by dosage (Table 4) and their low adjustments for regressions: P_total_ (R^2^ linear = 2 × 10^−6^; R^2^ quadratic= 0.0642) and P_total_ − P_resin_ (R^2^ linear = 0.0229; R^2^ quadratic = 0.1223).

The regression analysis showed adjustments for the levels of Ca, Mg, Si and P in the soil solution as a function of fertilization. They were significant for Mg and P (Table 4). Additionally, positive correlations between the concentrations of the nutrients present in greater quantities in thermophosphate in the soil solution corroborate the effect of the fertilizer on soil chemistry. Examples include correlations such as P_resin_ × Si (0.569; *p* = 0.001), P_resin_ × Ca (0.477; *p* = 0.009), P_resin_ × Mg (0.587; *p* = 0.007), Si × Ca (0.441; *p* = 0.017), Si × Mg (0.525; *p* = 0.003) and Ca × Mg (0.743; *p* < 0.000).

The levels of micronutrients in the soil did not differ significantly (Table 5). Furthermore, despite the good fits to the data for the quadratic responses found for Cu and Mn, the regressions were also not significant: B (R^2^ linear = 0.4002; R^2^ quadratic = 0.4005), Cu (R^2^ linear = 0.2639; R^2^ quadratic = 0.8017), Fe (R^2^ linear = 0.3412; R^2^ quadratic = 0.3428), Mn (R^2^ linear = 0.2228; R^2^ quadratic = 0.9786), and Zn (R^2^ linear = 0.0072; R^2^ quadratic = 0.5945).

### 2.2. Leaf Nutrient Contents

**Figure 1:**
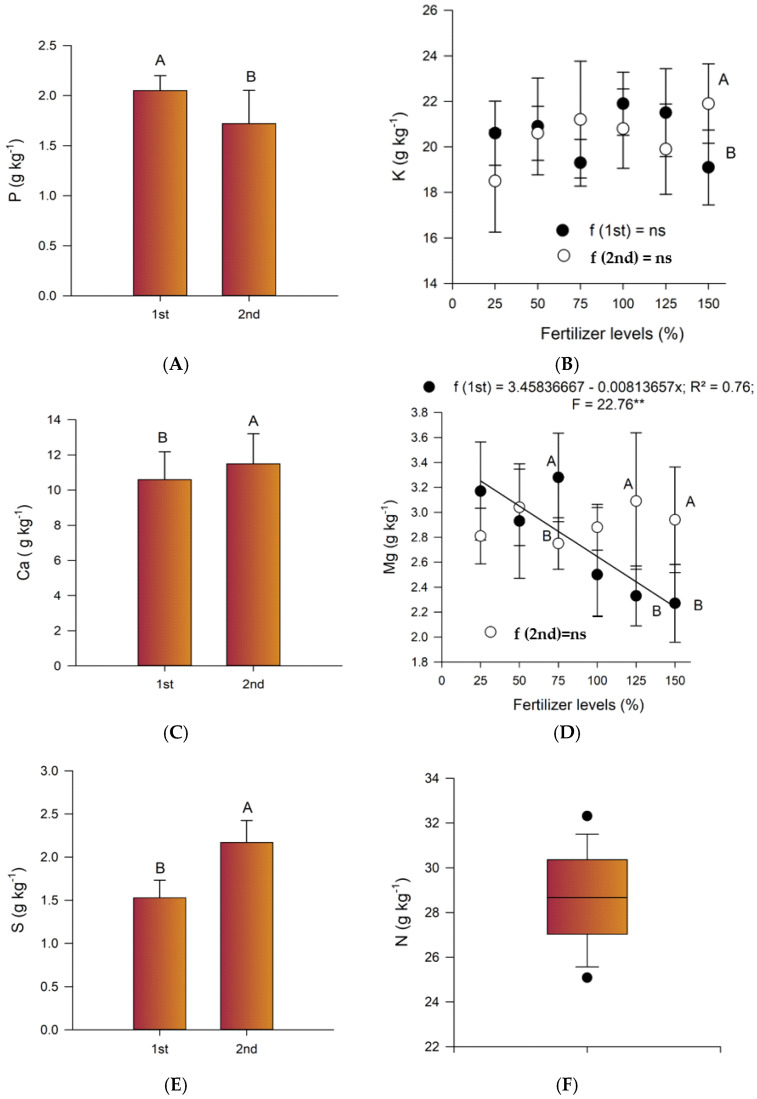
Macronutrients in ‘BRS SCS Belluna’ banana leaf tissue as a function of crop season (**A**,**C**,**E**) and/or thermophosphate fertilization (**B**,**D**). Average nitrogen value (**F**). Different uppercase letters for the crop cycle or the same level of thermophosphate application differ statistically from each other using the Tukey test (*p* < 0.05); ns: not significant; ** regression significant at 1% by F test; ● = first crop season; ○ = second crop season; vertical bars (I) = standard error.

In general, leaf contents for macronutrients were higher for the second crop season than for the first (Figure 1). P, Ca and S only had an effect on the cropping season (Table 6, Figure 1A,C,E). The content of leaf potassium was significantly differentiated by the interaction of the factors (Table 6, Figure 1B). The magnesium content in the leaf was a result of the levels of thermophosphate and the crop season, with a significant interaction between the factors in the first crop season (Figure 1D).

The data corroborate the possibility of dilution of the absorbed mineral due to greater phytomass production, since the dry matter of leaves produced in the first crop season was greater than that of the second (*p* = 0.0298, CV 21.8%). Leaf dry mass showed negative correlations with the concentration of six of the ten nutrients evaluated. On the other hand, the P content was higher in the first crop season than in the second (Figure 1A).

The nitrogen content in the leaf was not differentiated by the sources of variation (Table 6). Therefore, Figure 1F represents the nitrogen content regardless of the variation factors. The N content in the leaves was very similar to that found in the ‘Nam’ banana plant: 24.8 g/kg (first harvest) and 26.3 g/kg (second harvest) (Borges et al., 2006) [19], the former being the old nomenclature for the BRS SCS Belluna banana plant [17].

**Figure 2:**
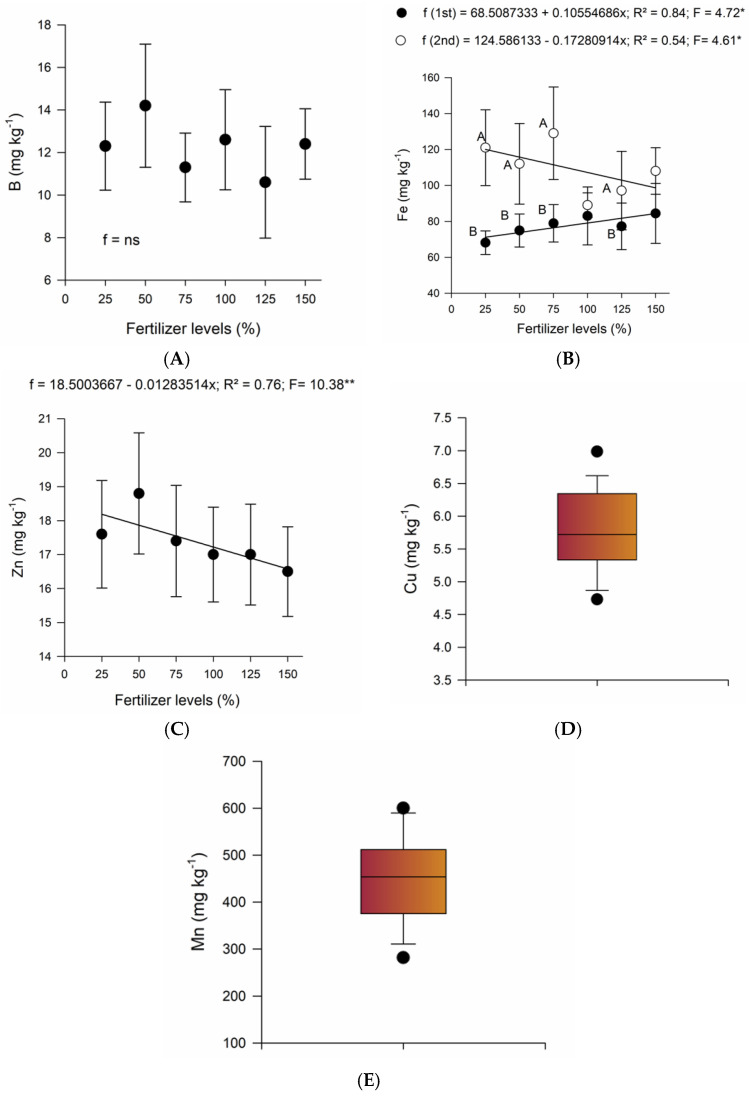
Micronutrients in ‘BRS SCS Belluna’ banana leaf tissue as a function of thermophosphate fertilization (**A**,**C**) and/or crop season (**B**). Average values for copper (**D**) and manganese (**E**). Different uppercase letters for the crop cycle or the same level of thermophosphate application differ statistically from each other using the Tukey test (*p* < 0.05); ns: not significant; ** regression significant at 1% by F test; * regression significant at 5% by F test; ● = first crop season; ○ = second crop season; vertical bars (I) = standard error.

B and Zn are the main micronutrients linked to deficiency symptoms presented by national banana farming [16,19]. An analysis of the leaf micronutrient data showed that fertilization with thermophosphate had an effect on B levels, although the regression analysis was not significant for B (Table 6). In light of these results, Figure 2A shows the average boron levels for each level of thermophosphate. The zinc content in the leaf had a significant effect depending on the levels of thermophosphate (Table 6) applied. Figure 2C shows the negative linear effect of thermophosphate fertilization on leaf zinc content.

Fe and Mn were the micronutrients with the greatest presence in leaf tissue (Table 6). In general, for the first crop season, the concentration of Fe increased linearly with the level of thermophosphate applied, while for the second season, the response was the opposite: the applications promoted a linear reduction in the concentration of foliar Fe (Figure 2B). When phosphate fertilizer levels of 25%, 50%, 75% and 125% were used, the Fe levels in the leaves were significantly higher in the second crop season (Figure 2B).

Borges et al. [19] reported higher foliar Fe levels in ‘Nam’ banana for the first crop season (128 mg kg^−1^) than for the second (68 mg kg^−1^).

Cu and Mn were not related to the sources of variation assessed in the leaf analysis (Table 6). Therefore, Figure 2D,E with a single bar represent the variation in the contents of these nutrients regardless of the variation factors.

The Cu content (Table 6, Figure 2D) was slightly below that observed in the Nam cultivar, 8.0 mg kg^−1^ (first harvest season) and 7.7 mg kg^−1^ (second harvest season). Among the micronutrients analyzed, it is the least required by banana plants [19].

The Mn content (Table 6, Figure 2E) was much higher than that found in ‘Nam’ banana, 183 mg kg^−1^ (first crop season) and 170 mg kg^−1^ (second crop season), by other authors. However, among all the nutrients evaluated, Mn had the highest coefficient of variation, ranging from 43 mg kg^−1^ to 574 mg kg^−1^, for the 24 banana genotypes evaluated, with averages of 285 mg kg^−1^ (first crop season) and 329 mg kg^−1^ (second crop season) [19].

**Figure 3:**
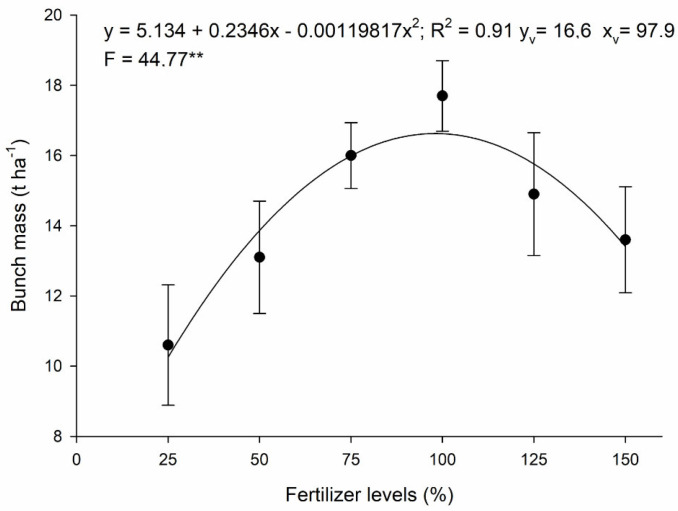
Cumulative bunch mass as a function of thermophosphate fertilization. ** significant at 1% by F test; vertical bars = standard deviation; CV = 10.6%.

The effect of thermophosphate fertilizer levels on bunch mass (Figure 3) shows a reduction in this indicator at the highest fertilizer levels, with a response at the optimum level of 97.9%.

**Figure 4:**
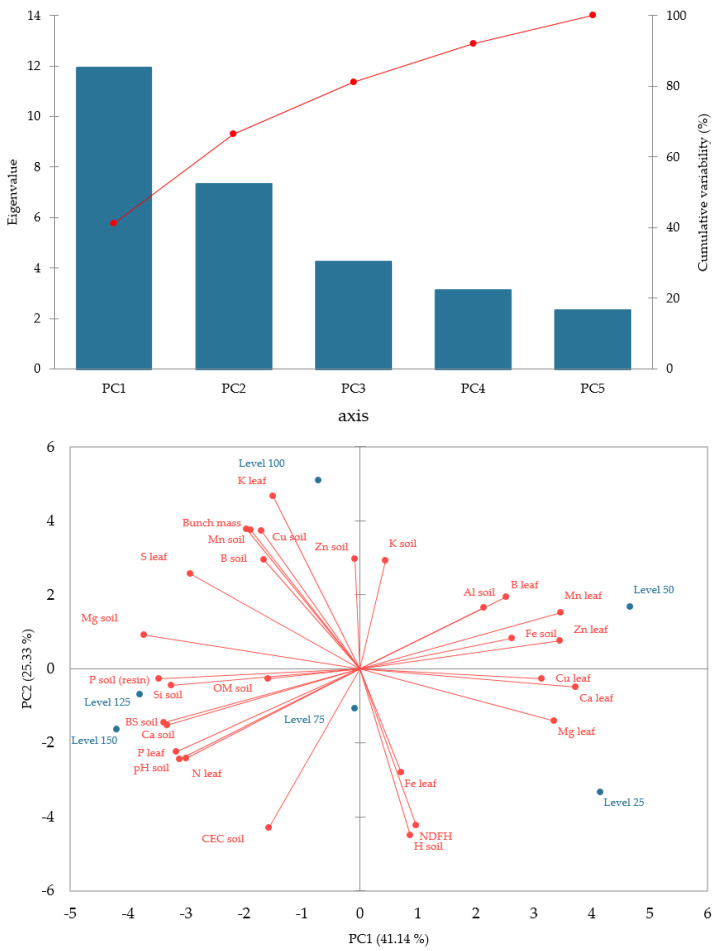
Biplot of the first two principal components and scree plot for the different components considered for the principal component analysis with greater eigenvalues.

**Figure 5:**
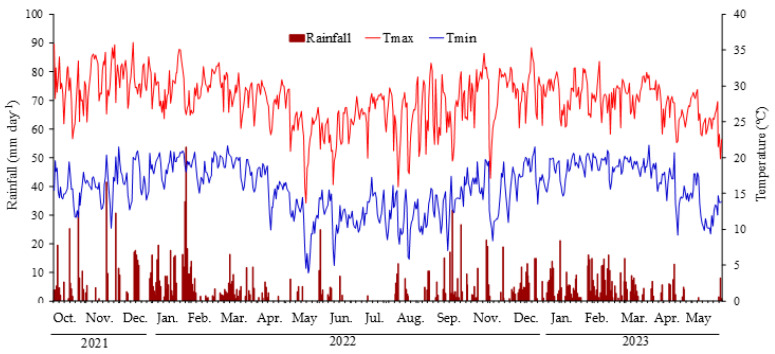
Maximum and minimum temperatures (°C) and rainfall (mm) in the months of October 2021 to May 2023 in the experimental area.

Principal component analysis (PCA) is used to obtain a small number of linear combinations of a set of variables that retain as much information on the original variables as possible [20]. The scree plot for the different components considered for the principal component analysis and the PCA biplot are shown in Figure 4. Principal components (PC1 and PC2) represented 66.47 per cent of the total variation in the data. PCI explained 41.14% of the total variation and effectively separated the contents of CEC, pH, BS, Si, P, Mg, B, Cu and Zn in the soil and N, P, S and K content in the leaves. The indicator that correlated with PC1 was bunch mass. Analyzing PC1 indicated that the values for this indicator were higher in relation to the values for the number of days from flowering to harvest (NDFH), the harvest season interval, which is on the opposite side of PC1. The lower temperatures and accumulated rainfall that occurred during the second harvest season (Figure 5) may be related to the higher NDFH and lower bunch mass.

PC2 accounted for 25.33% of the total variation and was mainly correlated with the levels of H, Fe, K in the soil and Fe, Mg, Ca, Cu, Zn, Mn and B in the leaves and NDFH. The PC2 analysis indicated that this separation occurred mainly due to the duration of the harvest season, which showed a negative correlation in the second growing season with bunch mass and, consequently, with yield. The PC2 scores and loadings indicated that the plants in this period produced the lowest bunch mass.

## 3. Discussion

Bananas grow quickly and accumulate high amounts of dry matter, thus requiring great amounts of nutrients. Nutrient demand varies between cultivars. Genetic differences are also found within genomic groups, due to the nutrient content of the soil and cultural practices. In addition, plants will find a balance with the nutritional status of the soil; i.e., the abundance of one nutrient can induce a deficiency of another.

Soil properties are interrelated, conditioning the availability of nutrients in the soil and being influenced by fertilization management and plant growth and yield. Organic matter (OM), for example, reflects the decomposition and stabilization of organic material deposited in the soil, contributing to mineral availability after mineralization and increasing loads available for exchange [3,21]. In this context, correlations showed positive effects for OM × CEC (0.449, *p* = 0.015), OM × SB (0.520, *p* = 0.004) and OM × BS% (0.487, *p* = 0.007). Phosphate fertilization could therefore have promoted more vigorous banana plants [22], and due to the high rates of production and dry matter cycling presented by the crop, it could have influenced the soil’s organic matter content [16]. Furthermore, phosphate fertilizer could have intensified these relationships. Li et al. [23] reported positive correlations between soil P available and dissolved organic carbon and microbial carbon.

The pH is associated with the availability of nutrients in the soil solution, interacting with CEC (due to pH-dependent loads) and BS% (due to the displacement of sites occupied by Al^3+^ and H^+^ ions). In fertilizer management, soil acidity can change due to the chemical reactions of fertilizers in the soil solution, the application of inadequate amounts or the nature of the nutrient loads present in the sources, which determine ionic exchanges between the plant and the soil solution, releasing H^+^ or OH^−^ ions. Moreover, acidity conditions the content of Al^3+^, which, above pH 4.0, decreases drastically as acidity decreases, reaching almost zero at pH 5.5 [3,21]. Therefore, thermophosphate could have potentially contributed to raising the pH due to its alkaline effect, although it did not have a significant impact on other variables, as found by other authors [11,12]. These relationships are particularly important for banana plants, which absorb large amounts of cationic nutrients, leading to acidification of the rhizosphere region and compromising their performance [13,14].

The means of the macronutrients present in the soil did not differ depending on the application of thermophosphate. The thermophosphate does not contain K in its composition, but does include Ca and Mg, which can be released into the soil solution [11,12]. On the lyotropic scale, both Ca^2+^ and Mg^2+^ bind more strongly to the colloidal charges of the soil than K^+^, potentially displacing K^+^ from the exchange complex and leading to its loss by leaching, especially in soils with predominantly sandy textures, such as that of the experimental area. A similar result could have occurred for S, which, despite not being present in the thermophosphate formulation, could compete for the charges present in the anion exchange complex with the P released by the thermophosphate and be lost through leaching [3,21]. However, the regressions for K and S did not show reduction trends for the concentration of these minerals in the soil.

In general, for banana plants, P is the macronutrient with the highest export rate in relation to the absorbed content [16]. In highly weathered tropical soils with low pH, phosphorus in the soil solution can form insoluble complexes with Fe and Al, making it unavailable for plant uptake [3].

The features of thermophosphate, such as its silicon (Si) content, pH correcting power and lower P solubility, may have contributed to the results (Table 3). In addition, the silicon present in the fertilizer (10%) can compete for phosphorus fixation sites. The positive correlation found between the content of these two minerals in the soil, P and Si, can support this finding and is in accordance with the results obtained by Pastore et al. [24] and Cartes et al. [25]. The pH correction effect of thermophosphate reduces the concentration of Al^3+^ ions in the solution, thereby decreasing the immobilization of phosphorus by these ions. Additionally, the gradual solubilization presented by thermophosphate reduces the amount of phosphorus available in the soil solution, which is then exposed to immobilization [3,21]. The correlations between P_resin_ and pH (0.524, *p* = 0.004) and P_resin_ and Al^3+^ (−0.380, *p* = 0.042) further support these hypotheses.

The most important minerals present in thermophosphate, in terms of quantity, are calcium (Ca), phosphorus (P), silicon (Si) and magnesium (Mg). P, present in a concentration of approximately 7.5% (equivalent to 17% P_2_O_5_) in thermophosphate, despite having a charge of the same nature as Si (−), exhibits much lower mobility and is less prone to leaching [3,21]. Additionally, the sorption relationship between these nutrients is asymmetric, with phosphorus interfering more with Si absorption than vice versa. This asymmetry may have contributed to the removal of Si from the system, either through leaching or absorption by plants [25]. These conditions may explain the significance and better fits found for the regression of P compared to those found for Si (Table 4).

In relation to Ca, a mineral present in greater quantities in thermophosphate (18% Ca), the effect of fertilization on its concentration in the soil solution may have been diluted by the annual application of limestone and thus may not have presented a significant value for the regression (Table 4). The limestone used, in addition to containing 25.7% Ca (36% CaO), when applied without incorporation, tends to accumulate mainly in the surface layer of the soil [21], enhancing the dilution effect. For Mg, the regression adjustment may have been significant (Table 4), as, despite being present in limestone, it is found in smaller quantities, 9.5% Mg (15.8% MgO). Therefore, the dilution effect on the Mg of thermophosphate is smaller. Furthermore, it is absorbed in smaller quantities by banana plants than calcium [16,26].

None of the micronutrients evaluated in the soil showed a significant difference in these contents. Except for Fe, these micronutrients are present in the composition of the thermophosphate, but in low quantities, so together, they make up approximately 1% of the fertilizer. Thus, a significant effect could have been obtained from larger doses of fertilizer. Fageria and Santos [11] managed to increase the levels of evaluated micronutrients (Cu, Fe, Mn and Zn) in soil cultivated with lowland rice after two production cycles and thermophosphate application rates. The significant results found by these authors may be due to the greater range between the P rates applied (299.9 kg P_2_O_5_ ha^−1^), in comparison to this study, that varied linearly by 20 kg P_2_O_5_ ha^−1^ for every application dose. Furthermore, according to Raij [27], except for B, all other micronutrients were present in the soil at initial levels (pre-planting) considered high, which makes finding significant responses difficult.

The leaf analysis showed variations only for N, Cu and Mn. N and K are the nutrients most absorbed by banana plants and the ones that are present in the highest concentrations in their leaf analysis, with much higher levels than the other mineral nutrients, and the higher or lower concentration between them can vary depending on the genotype [16,19].

Leaf macronutrient contents varied according to the cropping season, being higher in the second growing season than in the first (Table 6). This may have occurred due to the timing of bunch emission and seasonality, which, for banana plants, reduces leaf growth and emission during periods of low temperatures and precipitation, resulting in mineral dilution due to increased phytomass production in hot and rainy periods [16,21]. According to the climatological normal presented by Franco et al. [28] and Cunha and Martins [29], 71.1% of the bunches in the first season were emitted during the period of highest temperatures and precipitation (November to March), while for the second season, the emission in the same period was 51.4%. Furthermore, in the coldest and driest months, from May to August, emissions were higher for the second crop season (16.9%) than for the first (12.5%). The results obtained by Costa et al. [30], which considered the effect of seasonality on the leaf content of banana plants, showed that leaf samples of the Prata and Grand Naine cultivars collected in winter had higher nutrient content compared to those in summer.

The higher concentration of P in the first growing season can be explained by the fact that its contact with the roots depends almost exclusively on diffusion, unlike the other nutrients [2,3,21]. The increased availability of water in the soil in the months with the highest rainfall may have contributed to the contact between the P and the banana roots. Furthermore, the greater quantity of the total P_2_O_5_ present in thermophosphate is soluble in citric acid. The greater growth of banana plants during the hottest and rainiest period can release a greater amount of H^+^ into the soil solution, acidifying the rhizosphere and contributing to the solubilization of the phosphorus present in the fertilizer. For the leaf tissue of the Nam cultivar, Borges et al. [20] reported a content of 1.6 g P kg^−1^ (first and second cycles), with averages of 1.4 g P kg^−1^ and 1.6 g P kg^−1^ for the first and second crop seasons, respectively, in 24 genotypes evaluated. For banana trees from the Cavendish subgroup cultivated in the state of São Paulo, Brazil, Teixeira et al. [31] establish optimal leaf P levels between 1.7 g kg^−1^ and 2.7 g kg^−1^. Thus, the result, 1.89 g P kg^−1^ (Table 6), is in agreement with these studies.

B and Zn showed different levels in the leaves according to the thermophosphate fertilization. For the Nam cultivar, Borges et al. [19] reported leaf contents of 35.0 mg B kg^−1^ (first cycle), 29.3 mg B kg^−1^ (second cycle), 28.0 mg Zn kg^−1^ (first cycle) and 15.7 mg Zn kg^−1^ (second cycle). Although B is antagonistic to Ca, this effect was not observed even with the supply of Ca from the application of higher levels of thermophosphate (Table 6). The reduction in leaf Zn as a function of the increase in the level of thermophosphate applied (Figure 1B) may have been caused by the increase in P, with which Zn presents antagonism [16,21]. P-Zn antagonism can be explained by P-Zn interactions in the soil itself, dilution of the Zn absorbed by the plant due to increased biomass production (dilution effect) in response to P application, reduced zinc absorption and/or translocation influenced by the addition of P, and P interference in Zn utilization by the plant. Sometimes, higher levels of PO_4_^3−^ ions can also reduce the colonization of mycorrhizal fungi, which leads to a reduction in the absorption surface of the roots. Inadequate long-term applications of P fertilizers result in the progressive accumulation of P in the soil and show that if the application of Zn is not properly considered or Zn is not applied at the optimum time, a serious Zn deficiency can occur [32].

Due to their high concentration in soils, Fe and Mn are not linked to deficiencies in Brazilian banana plantations, and among the micronutrients recommended for the nutritional diagnosis of banana trees, they are those that present the highest concentrations in leaf tissue [16,19].

In general, the leaf contents were within the ranges considered optimal for cultivars of the Cavendish subgroup in the state of São Paulo, according to Teixeira et al. [31]. K (20.5 g kg^−1^) was the nutrient that showed the greatest deviation from the optimal lower limits presented by these authors, which were 30 g kg^−1^, 35 g kg^−1^ and 27.9 g kg^−1^. Less-than-optimal foliar potassium can be explained either by inadequate fertilization or by competitive interactions with Ca and Mg. K, Ca and Mg utilize the same or similar absorption mechanisms, which leads to competition. High levels of K in the soil can reduce the availability of Mg for plants, leading to Mg deficiency. The balance of these cations in the soil can have a significant impact on plant growth and development. Soil acidity and high concentrations of other cationic nutrients, such as K and Ca, can contribute to K-induced Mg deficiency in certain regions. The proportion of K, Ca and Mg in the soil is crucial for optimal plant nutrition. For example, a high K/Mg ratio can have a negative impact on Mg absorption [33]. The antagonistic effect of potassium (K) on magnesium (Mg) is greater when compared to the effect of Mg on K in root absorption and transport within plants. This indicates that balanced use of K and Mg fertilizers is necessary to maintain high levels of Mg available to plants and to mitigate K-induced Mg deficiency, particularly in plant species with high K requirements or in soils with high K availability. The relationship between Mg and K in plant tissues can be either antagonistic or synergistic, depending on factors such as the plant species, cell type, leaf age and location of the source and sink organs. Synergistic effects of K and Mg have been observed in photosynthesis, carbohydrate transport and distribution, nitrogen metabolism, and turgor regulation. It is desirable to define optimal K/Mg ratios for soils and plant tissues to maintain adequate nutritional status in plants and support agricultural production [34].

Fast-growing banana plants need a significant amount of nutrients, mainly potassium (K) and nitrogen (N), for proper development. These nutrients are directly related to the development of the plant and the production and quality of the fruit, and the amount extracted differs depending on the cultivar, the phenological state and the age of the plant [35]. Oliveira et al. [35] evaluated soil parameters that limit banana plant growth and development in Vale do Ribeira, São Paulo State, Brazil, and concluded that, although the average nutrient concentrations in the soil were considered high, the K, Mg and S levels in the soil failed to provide adequate banana plant nutrition, as these nutrients were deficient in the plants.

The results obtained in this study indicated optimal levels of Ca and Mg in the soil and leaves and lower K levels in the leaves which were not related to fertilization with increasing levels of thermophosphate. Besides competitive interaction between K, Ca and Mg, another probable reason is that the levels of potassium that should have been applied in the fertilizations were underestimated. According to Teixeira et al. [36], the official reference prior to this study, according to the K contents in the soil, the recommendations were as follows: planting (soil K = 1.08 mmol dm^−3^ and recommendation = 310 kg ha^−1^); top dressing (soil K = 4. 61 mmol dm^−3^ and recommendation = 150 kg ha^−1^); summer 2020–2021 (soil K = 3.53 mmol dm^−3^ and recommendation = 150 kg ha^−1^); summer 2021–2022 (soil K = 3.47 mmol dm^−3^ and recommendation = 150 kg ha^−1^). The soil K is lower than the banana plant requirement for the state of São Paulo, which was adjusted according to Teixeira et al. [37]; after the start of this study, the updated recommendations were 600 kg ha^−1^ with soil K = 1.08 mmol dm^−3^ and the same values of 150 kg ha ^−1^ for the other fertilizations. As a result, 290 kg ha^−1^ of K was used in this study, practically 50% less than the updated recommendation.

In summary, fertilization with different levels of thermophosphate had no influence on soil chemical properties and the contents of K and S in the soil. Ca and Si exhibit linear adjustments of 64.0% and 69.7%, respectively, depending on the levels of thermophosphate fertilization, but they are not significant. Mg and P contents increased linearly because of fertilization with thermophosphate, with regression equations adjusted to 93.6% for Mg and 80.8% for P.

Fertilization with thermophosphate does not affect the levels of micronutrients in the soil. The application of fertilizer primarily decreases the leaf Zn content. Fe and Mg depend on the level of thermophosphate application. The crop season influenced leaf contents of P, Ca and S and interacted with the fertilizer application levels for leaf concentrations of Fe, Mg and K. N, Cu and Mn in leaf tissue are not influenced by any of the variation factors evaluated, level of thermophosphate fertilization and crop season. The leaf P content can be considered adequate.

Increasing the levels of thermophosphate influenced the content of macro- and micronutrients in the soil and leaf tissues. Phosphate fertilization promoted banana plants with greater bunch mass and, due to the crop’s high production rates and dry matter cycling, may have influenced the soil’s organic matter content.

Phosphorus is essential for the processes of photoassimilation, storage and transfer in plant tissues. However, excess P promotes rapid plant growth and can affect the availability of other nutrients, impairing their development, which explains the reduction in bunch mass yield with higher levels of thermophosphate.

Principal components 1 and 2 could clarify 41.5% and 24.9% of the total variance, respectively. Relationships among bunch mass, soil and leaf nutrients can be detected using the loading plot of PCA. The recommended level of fertilization (100%) contributed to the separation of bunch mass, K and S in the leaf and Cu, Zn, Mn, B and Mg in the soil. The 125% and 150% levels are highly positively correlated with the highest levels of Si, P, Mg and Ca in the soil and higher pH, P and leaf N. The 25% level was more closely related to high H content in the soil (active acidity) and a longer period between the emission of the inflorescence and the harvest (NDFH) and leaf Fe. High Fe content in the soil, among other factors, is positively related to soil acidity.

The PCA showed a correlation between the levels of thermophosphate applied (75, 125 and 150% of the recommended level) and the organic matter content in the soil. Possible hypotheses about this result can be explained on the basis that the relationship between organic matter and soil P is complex and dynamic. Understanding this relationship is crucial to optimizing P availability for plant growth and managing the P cycle on farms [38]. Soil organic matter acts as both a source and a sink for P in the soil. Although it can mineralize P and release it into the soil solution, it can also bind it, making it less available to plants. The processes of P retention in soils, in connection with the presence of OM, has been a topic debated for years without clear conclusions. In principle, the competitive relationship between OM and soil P should increase P availability as a result of blocking sorption sites for P and OM constituents [39]. Similarly, with increasing levels of thermophosphate, an increase in soil P availability is to be expected, which increases P absorption sites to the detriment of OM constituents. Consequently, there may have been an increase in the soil’s OM content. The quantity and quality of organic matter and the conditions in which P sorption occurs may also play a decisive role in this process, and this relationship is highly dependent on environmental conditions [40]. In addition, soil organic matter can form complexes with P, influencing its mobility and availability in the soil, and constituents of organic matter, such as humic and fulvic acids, can bind to P, affecting its availability and retention in the soil [39,40].

These results, obtained in two consecutive harvesting seasons with different climatic conditions, provide additional information on the role of phosphorus dynamics in the soil and in the leaves as well as its correlation with the duration of the harvest season and the yield of bunch mass and could be useful for subsidizing fertilization programs aimed at a sustainable approach to phosphorus nutrition in banana fields.

The study examined the role of phosphorus fertilization on banana plantations, with the aim of improving crop quality, increasing productivity and ensuring the long-term sustainability of these plantations. Given the limited availability of phosphate resources, optimizing phosphorus fertilization is a continuous process that is crucial for future research.

## 4. Materials and Methods

### 4.1. Site Description and Crop Management

The experiment was performed in an experimental area of the São Paulo State University (UNESP) College of Agricultural Sciences, located in the city of São Manuel, state of São Paulo, Brazil (22°77′ S; 48°34′ W and 740 a.s.l.). According to the Köppen–Geiger classification, the climate of the area is type Cfa, that is, a hot temperate climate (mesothermic), with concentrated rains from November to April (summer) and an average annual rainfall of 1376.70 mm; the mean temperature of the hottest month exceeds 22 °C [29]. The soil of the experimental site is classified as Dystrophic Typic Hapludox [41].

Before the banana seedlings were planted, the experimental area was covered with a mix of green manure species, which were incorporated into the soil along with the preparation, plowing and harrowing operations. Pre-planting soil analysis for the 0–20 cm deep layer showed the following: 5.4 pH (CaCl_2_), 11 mg OM dm^−3^ (OM, organic matter), 9 mg P_resin_ dm^−3^ (P, phosphorus), 2 mg S dm^−3^ (S, sulfur), 32 mg Fe dm^−3^ (Fe, iron), 2.4 mg Cu dm^−3^ (Cu, copper), 8.5 mg Mn dm^−3^ (Mn, manganese), 2.2 mg Zn dm^−3^ (Zn, zinc), 0.19 mg B dm^−3^ (B, boron), 0 mmol_c_ Al^3+^ dm^−3^ (Al^3+^, exchangeable aluminum), 15 mmol_c_ Al^3+^ + H dm^−3^ (Al^3+^ + H total acidity), 1.08 mmol_c_ K dm^−3^ (K, potassium), 16 mmol_c_ Ca dm^−3^ (Ca, calcium), 6 mmol_c_ Mg dm^−3^ (Mg, magnesium), 23 mmol_c_ dm^−3^ base sum (SB), 38 mmol_c_ dm^−3^ cation exchange capacity (CEC), 60% base saturation (BS%), 843 g dm^−3^ (sand), 121 g dm^−3^ (clay) and 36 g dm^−3^ (silt).

The soil was prepared by plowing and harrowing 60 days before the seedlings were transplanted. The soil was corrected to increase the base saturation to 70%, a procedure that was carried out in the following cycles when necessary, according to the soil analysis [35]. Agricultural limestone from the manufacturer Horical^®®^ (Chennai, India) was used, with 36% CaO and 15.8% MgO. Potassium (K) was supplied as potassium chloride, nitrogen (N) was supplied as urea and ammonium sulfate was used to provide a minimum of 30 kg S ha^−1^ year^−1^. The following rates were applied: 275 kg K ha^−1^ and 190 kg N ha^−1^ (side dressing), 125 kg K ha^−1^ and 190 kg N ha^−1^ (summer 2020/21), and 125 kg K ha^−1^ and 190 kg N ha^−1^ (summer 2021/22). P was supplied as powdered thermophosphate (Yoorin Master 1, Yoorin^®^, Poços de Caldas, Brazil), which, in addition to phosphorus (17.5% P_2_O_5_), contains 18% Ca, 10% silicon (Si), 7% Mg, 0.55% Zn, 0.3% Mn, 0.1% B and 0.05% Cu.

Planting was carried out in furrows with a spacing of 2.0 m between rows and 2.5 m between plants (2000 plants ha^−1^). Seedlings of the BRS SCS Belluna cultivar were produced by micropropagation [42], acclimatized in the nursery, and planted in the field when they had 5 to 6 leaves and were approximately 30 cm tall.

A replicated trial was carried out during two harvest seasons. The first harvest season began in October 2021 and extended throughout 2022, and the second began in the second half of 2022 and ended in May 2023. Daily rainfall (mm) and maximum, minimum and average temperatures (°C) were measured during the entire experimental period (Figure 5).

Fertilizations (Table 7) were distributed over the period of greatest rainfall stability and precipitation volume, from November to February [28,29].

Weed control, tiller thinning, dry and diseased leaf removal, pest and disease control, male inflorescence elimination, pistil removal and harvesting were performed according to recommended practices for the crop [16,43].

### 4.2. Treatments and Experimental Design

According to the soil analysis and a potential yield of less than 20 t ha^−1^ [31], a fertilization pattern of 100% phosphorus (P) was defined using treatments corresponding to levels of P, i.e., 25%, 50%, 75%, 100%, 125% and 150% of the recommended level for the crop [16,43]. These levels were calculated based on the P_resin_ content in the soil at a depth of 0–20 cm. For planting and top-dressing fertilization, pre-planting soil sampling was conducted across the experimental area. For fertilization, soil collections were carried out in the plots that received the reference level (100%).

Thermophosphate was considered an independent variable and was evaluated at different application levels, which were subdivided based on the reference level corresponding to 100% of the P_2_O_5_ recommended by Teixeira et al. [43] (Table 7).

The experimental design included randomized blocks with five replicates with six plants per plot, plus guard plants outside the trial. The plots were represented by the level of thermophosphate application (Table 7). For nutritional leaf diagnosis, a randomized block experimental design was used in a double factorial scheme: thermophosphate application levels (1st factor) and crop season (2nd factor).

### 4.3. Soil and Leaf Analysis

Soil chemistry was evaluated for P, K, Ca and Mg (resin); P_total_ (nitro perchloric); Cu, Mn, Fe and Zn (DTPA); B (hot water); Al^3+^ and H (KCl); S (calcium phosphate); pH and Si (CaCl_2_); organic matter (oxidation); and sum of base (SB), CEC and BS% (calculated). The SB was calculated by the sum of the bases Ca, Mg and K. The CEC was calculated from the sum of the K, Ca, Mg, H and Al, and the BS% by the relationship between the SB and the CEC [27]. Storage for unavailable P was calculated by the difference between P_total_ and P_resin_. Soil sampling for chemical analysis was carried out in the fertilizer line and four months after the application of the last dose of fertilizer 2021/22, in June 2022. Thus, five samples (0–20 cm depth) were collected per plot and were homogenized to form a composite sample; in total, five composite samples were collected for each level of thermophosphate application.

The N, P, K, Ca, Mg, S, B, Cu, Fe, Mn and Zn mineral contents in leaf tissue were analyzed according to the methodology proposed by Malavolta et al. [44]. Tissue sampling followed the collection recommendation according to the assessment for leaf diagnosis. Thus, for the 1st and 2nd crop seasons, and for the 3rd leaf from the apex of the banana plant, a section approximately 10 cm wide was sampled in the intermediate part of the limb and on each side of the central vein [16].

### 4.4. Harvesting, Bunch Mass and Harvest Season Assessments

Banana plants were assessed at the end of each growing season, when the bunches were harvested. The banana bunches were harvested in the morning when the fruit in the middle of the second hand had reached a minimum size of 34 mm in diameter [43]. The banana yield was estimated from the fresh mass of marketable bunches per plant. The cumulative bunch mass corresponded to the sum of the two harvesting seasons evaluated. The time duration of the harvest season was measured by counting the number of days between flowering and harvest.

**Figure 6:**
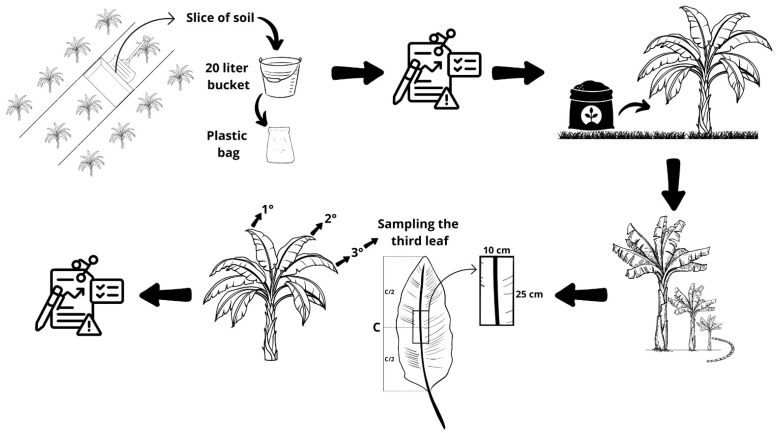
Overview of the experiment.

Figure 6 shows the general overview of the experiment.

### 4.5. Statistical Analysis

The nutritional components were analyzed using the Shapiro–Wilk normality test and analysis of variance. When the F test was significant (*p* < 0.05), the means were compared using Tukey’s test. The analyses were carried out using the statistical program R, version 4.3.1 [45]. Additionally, linear and quadratic regressions were used to identify the performance of the data in relation to the application levels of the thermophosphate. Principal component analysis was carried out using XLSTAT Premium software (version 26.4.1, Addinsoft, New York, NY, USA) [46] to better visualize and explain the variability between the yield and seasonality data and the nutrient contents in the soil and leaves assessed.

## 5. Conclusions

Fertilizing BRS SCS Belluna banana plants with thermophosphate had a nutrient-dependent effect on soil fertility attributes and nutrient levels in the leaves, which had an effect on the plant’s growth and production. The level of phosphate fertilization of 25% of the recommended level was positively related to a longer time between flowering and harvest. The application of 97.9% of the recommended level of phosphorus led to the highest yield of bunch mass. The greater availability of water in the soil in the months with the highest rainfall may have contributed to an increase in the levels of P and other nutrients and, consequently, to an increase in bunch mass and a reduction in the harvest season duration.

## Figures and Tables

**Table 1:** Chemical soil properties (0–20 cm depth).

Fertilization Levels	OM (g dm^−3^)	pH *	Al^3+^ (mmol_c_ dm^−3^)	H (mmol_c_ dm^−3^)	CEC (mmol_c_ dm^−3^)	BS (%)
25%	7.07	4.65	1.35	17.9	38.6	49.2
50%	7.39	4.42	2.37	17.3	37.3	47.0
75%	8.38	4.67	1.58	17.1	38.9	50.9
100%	7.71	4.59	1.55	16.2	37.1	49.8
125%	6.78	4.82	0.99	17.6	38.2	50.7
150%	9.03	4.71	1.69	17.3	39.3	50.9
*p*-value	0.4971	0.6815	0.6086	0.6871	0.9560	0.9812
Average	7.73	4.64	1.59	17.2	38.2	49.7
CV (%)	12.4	3.30	25.1	5.27	6.73	11.8

OM = organic matter; CEC = cation exchange capacity; BS = base saturation; CV = coefficient of variation. * pH (1:2.5 soil/CaCl_2_ suspension 0.01 mol L^−1^).

**Table 2:** Macronutrients and silicon in soil (0–20 cm) available for plant uptake.

Fertilization Levels	K (mmol_c_ dm^−3^)	Ca (mmol_c_ dm^−3^)	Mg (mmol_c_ dm^−3^)	S (mg dm^−3^)	Si (mg dm^−3^)
25%	2.86	11.9	2.67	3.31	4.20
50%	2.64	11.9	3.04	5.08	4.55
75%	3.08	13.1	3.33	4.02	4.42
100%	3.47	11.6	3.42	4.48	4.48
125%	2.46	13.6	3.55	3.31	5.42
150%	2.70	13.8	3.89	4.90	5.14
*p*-value	0.4597	0.9497	0.2271	0.0804	0.8757
Average	2.87	12.5	3.32	4.18	4.70
CV (%)	14.4	18.9	11.8	13.4	17.5

CV = coefficient of variation.

**Table 3:** Phosphorus fractions in soil (0–20 cm depth).

Fertilization Levels	P_resin_	P_total_	P_total_ − P_resin_
	(mg dm^−3^)		
25%	7.56	237.2	229.7
50%	6.99	239.4	232.4
75%	6.69	216.6	209.9
100%	9.64	268.4	258.8
125%	12.1	237.0	225.0
150%	13.5	228.2	214.5
*p*-value	0.3249	0.6048	0.5962
Average	9.40	237.8	228.4
CV (%)	29.7	8.86	9.06

P_total_ − P_resin_ = estimate of unavailable phosphorus; CV = coefficient of variation.

**Table 4:** Regression equation and adjustment for soil analysis (0–20 cm) as a function of fertilization.

Nutrient	Equation	R^2^	F Value
Ca	y = 10.8620000 + 0.01894857x	0.6408	0.94
	y = 10.9970000 + 0.01489857x + 0.00002314x^2^	0.6421	>0.00
Mg	y = 2.54266667 + 0.00884571x	0.9558	7.21 *
	y = 2.40700000 + 0.01291571x − 0.00002326x^2^	0.9646	0.24
Si	y = 3.96440000 + 0.00841829x	0.6970	1.15
	y = 4.15040000 + 0.00283829x + 0.00003189x^2^	0.7103	0.03
P	y = 4.63693333 + 0.05443886x	0.8084	4.78 *
	y = 8.09160000 − 0.04920114x + 0.00059223x^2^	0.9359	0.68

* = significant at 5% by F test.

**Table 5:** Micronutrients in soil (0–20 cm depth) available for plant uptake.

Fertilizer Levels	B (mg dm^−3^)	Cu (mg dm^−3^)	Fe (mg dm^−3^)	Mn (mg dm^−3^)	Zn (mg dm^−3^)
25%	0.14	2.42	28.6	5.37	2.56
50%	0.16	3.10	29.8	6.90	2.58
75%	0.15	2.96	26.3	7.50	2.73
100%	0.18	3.19	27.5	8.13	3.09
125%	0.15	3.21	28.5	7.75	3.00
150%	0.19	2.88	25.8	6.50	2.12
*p*-value	0.3046	0.3248	0.8587	0.5364	0.7018
Average	0.16	2.96	27.7	7.02	2.68
CV (%)	13.0	10.4	10.8	18.1	19.6

CV = coefficient of variation.

**Table 6:** The analysis of variance for the effects of the levels of thermophosphate fertilization (TF) and the crop season (CS) on the nutrient content in the leaf of ‘BRS SCS Belluna’ banana.

Factors	N	P	K	Ca	Mg	S
	F value					
TF	2.25	1.23	1.07	2.28	3.14 *	0.79
CS	0.25	27.4 **	0.03	5.40 *	4.53 *	117.1 **
TFxCS	0.77	1.04	2.85 *	0.56	6.12 **	2.04
	(g kg^−1^)					
Average	28.5	1.89	20.5	11.0	2.83	1.85
CV (%)	4.25	6.78	4.97	7.36	6.10	6.36
	B	Cu	Fe	Mn	Zn	
	F value					
TF	3.28 *	1.05	1.69	2.32	3.64 **	
CS	2.55	2.61	67.7 **	2.73	1.59	
TFxCS	0.59	1.56	3.54 **	2.08	1.85	
	(mg kg^−1^)					
Average	12.2	5.78	93.6	440	17.4	
CV (%)	9.08	5.80	7.86	10.6	4.03	

** significant at 1% by F test; * significant at 5% by F test; CV: coefficient of variation.

**Table 7:** Thermophosphate levels calculated based on the P_2_O_5_ fertilization recommendation [35].

Fertilization/Levels	25%	50%	75%	100% *	125%	150%
	kg ha^−1^					
Planting	57	114	171	229	285	343
Top dressing	57	114	171	229	285	343
Summer 2020/21	114	229	343	457	571	686
Summer 2021/22	72	143	215	286	358	429

* recommended level [43].

## Data Availability

Data are contained within the article.
